# Preference of Menstrual Hygiene Products and Their Usage Among Medical Students: A Cross-Sectional Study in Chengalpattu District

**DOI:** 10.7759/cureus.82560

**Published:** 2025-04-19

**Authors:** Divyaparvathy J, Harini Raj Shankar Raj, Ardra Merin George, Venkatesh Karthikeyan

**Affiliations:** 1 Community Medicine, Tagore Medical College and Hospital, Chennai, IND; 2 Research, Tagore Medical College and Hospital, Chennai, IND; 3 Community and Family Medicine, All India Institute of Medical Sciences, Patna, Patna, IND

**Keywords:** health education & awareness, menstrual cups, menstrual health, menstrual hygiene products, sanitary pads

## Abstract

Background: In India, millions of women still face significant barriers to safe menstrual hygiene management. This study focuses on medical students to assess their preferences for various menstrual hygiene products.

Methodology: This cross-sectional study was conducted at a private medical college and hospital in Chengalpattu district among undergraduate medical students and postgraduates. The estimated sample size was 348, and data were collected using a semi-structured questionnaire. The data were entered into Microsoft Excel (Microsoft Corporation, Redmond, Washington) and analyzed using IBM SPSS Statistics for Windows, Version 24 (Released 2016; IBM Corp., Armonk, New York).

Results: The mean age at menarche among study participants was 13 years (SD ±1.37), with the range spanning from 9 to 13 years. Of the 351 participants, 94 (26.8%) reported irregular cycles, while the remainder had regular menstrual cycles. Among the participants, 93.7% used sanitary pads, 3.4% used menstrual cups, and 1.4% used tampons or reusable pads.

Conclusion: Ensuring access to clean washroom facilities reserved for staff and scheduling breaks for menstrual hygiene needs can help maintain proper menstrual hygiene. Promoting awareness about menstrual cups is crucial to encourage their adoption and to empower individuals with a sustainable and effective menstrual hygiene option.

## Introduction

India is the second-most populous country in the world, with over 1.4 billion people. With a significant portion of the population being women and girls of reproductive age, the number of menstruating individuals is substantial [1]. India comprises more than 355 million women and girls who are menstruating. However, millions of women still face significant barriers to safe menstrual hygiene management. Menstruation in India is often surrounded by cultural taboos and myths, which can lead to stigmatization and secrecy. These cultural factors influence how menstruation is perceived and managed [2]. According to the National Family Health Survey 5 (NFHS-5), there has been a rise in hygiene product usage in women aged 15-24 years, from 58% in NFHS-4 (2015-2016) to 78% in NFHS-5 (2019-2021). Among this population, 77% use disposable hygiene products like sanitary napkins and tampons, while only 0.3% use reusable products like menstrual cups [3].

Sanitary pads offer reliable protection against menstrual flow and provide comfort and ease of use without the need for insertion. However, they are not necessarily ideal, as they have been associated with leakage, chafing, vaginal discharge, and reproductive tract infections [4,5]. In contrast, menstrual cups are more affordable, durable, and can collect more blood than sanitary pads [6]. They are also eco-friendly and reduce the large amount of waste generated by other disposable hygiene products [7]. Yet they are characterized by certain barriers, including difficulties with insertion and removal, higher initial cost of purchase, and lack of awareness [8]. This study focuses on undergraduate and postgraduate medical students to assess their preference for various menstrual hygiene products. With an intense medical school schedule, long hours of studying, clinical rotations, and exams, it can be difficult to find time for proper menstrual hygiene practices, like frequent pad changes or cleaning. In addition, limited access to clean bathrooms, especially during clinical rotations in hospitals, can be a major obstacle. The main objectives of the study were to assess the preference of menstrual hygiene products and their usage among medical students of a tertiary care hospital in the Chengalpattu district, and to identify the bottlenecks and the key factors influencing product choice, including accessibility, affordability, awareness, and perceived comfort in the usage of menstrual hygiene products during menstruation.

## Materials and methods

This institution-based cross-sectional study was conducted at a private medical college and hospital in Chengalpattu district, with the aim of evaluating menstrual hygiene practices among female medical students. The study population comprised female medical students of reproductive age who were studying in that institute. The study was carried out from January 2024 to June 2024.

Study participants

The study included all female medical students within the reproductive age group (18-45 years) who were enrolled in MBBS or postgraduate programs and were willing to participate in the study. Participants were selected using a non-random convenience sampling method. Informed consent was obtained from each participant, ensuring they understood the purpose and nature of the study. All participants who were willing to take part in the study were considered.

Sample size calculation

The sample size was determined based on a previous study by Ramsay et al., which reported a prevalence of 32% for the usage of menstrual hygiene products. Considering this prevalence, a 95% confidence level, an absolute error of 5%, and a non-response rate of 20%, the minimum sample size required for the study was estimated to be 348 participants [9].

Data collection

Data were collected using a semi-structured questionnaire designed specifically for this study. Before the start of data collection, the questionnaire and overall study framework were reviewed by a panel of experts in the fields of reproductive health, epidemiology, and biostatistics. Based on the expert reviews and opinions, minor modifications were made to the questionnaire for better clarity. A pilot study was conducted with 30 samples and processed to identify any flaws in the study tool. Suggestions from the pilot group and experts were incorporated to improve the question structure. The pilot study participants were not included in the main study. The questionnaire was administered through Google Forms, allowing participants to provide responses online. The questionnaire was divided into two major sections. The first section included socio-demographic information, such as the participant's age, year of study, and other relevant demographic factors. The second section addressed menstrual hygiene practices, including the participants’ preferences regarding menstrual hygiene products, the type of product used (e.g., sanitary pads, menstrual cups, tampons), the factors influencing product choice (such as comfort, accessibility, cost, and environmental concerns), and their general knowledge and practices related to menstrual hygiene. Efforts were made to ensure the accuracy and reliability of the collected data through data cleaning and screening to check for incomplete entries, duplicate submissions, and logical inconsistencies.

Data management and analysis

The collected data were systematically entered into a Microsoft Excel spreadsheet (Microsoft Corporation, Redmond, Washington) for organization and cleaning. Descriptive statistical analyses were performed using IBM SPSS Statistics for Windows, Version 24 (Released 2016; IBM Corp., Armonk, New York). Descriptive statistics such as frequencies, percentages, means, and standard deviations were used to summarize socio-demographic characteristics and menstrual hygiene practices. Associations between various factors and menstrual hygiene product use were also assessed.

Ethical considerations

The study was conducted in adherence to ethical guidelines, with confidentiality maintained for all data collected. Participants were assured that their responses would be anonymous and used solely for the purpose of the study. The study protocol was approved by the Institutional Ethics Committee of the medical college.

## Results

The study encompassed 351 female medical students and doctors, with a mean age of 21.4 (±2.59) years and an age range of 17 to 33 years. The sociodemographic profile of the study participants is presented in Table 1. Monthly income, occupation of the father and mother, and educational qualifications of both parents were assessed according to the modified Kuppuswamy scale.

**Table 1:** Sociodemographic profile of study participants (N=351)

Variables	Frequency (n)	Percentage (%)
Monthly family income		
Class I – Upper class	9	2.6
Class II – Upper-middle class	22	6.3
Class III – Middle class	148	42.1
Class IV – Lower-middle class	83	23.6
Class V – Upper-lower class	56	15.9
Class VI – Lower-lower class	22	6.3
Class I VII – Very low class	11	3.1
Educational qualification of the mother		
Illiterate	6	1.7
Primary school	6	1.7
Secondary school	18	5.2
High school	44	12.8
Diploma/intermediate	49	14.2
Graduate/post-graduate	168	48.8
Professional/honors	53	15.4
Educational qualification of the father		
Illiterate	7	2.0
Primary school	2	0.6
Secondary school	18	5.3
High school	34	9.9
Diploma/intermediate	40	11.7
Graduate/postgraduate	170	49.7
Professional/honors	71	20.8
Occupation of the mother		
Unemployment	94	29.6
Clerical/farmer/shop owner	11	3.5
Semi-skilled	15	4.7
Skilled	40	12.6
Semi professional	47	14.8
Professional	111	34.9
Occupation of the father		
Clerical/farmer/shop owner	39	11.7
Professional	195	58.7
Semi professional	41	12.3
Semi-skilled	5	1.5
Skilled	48	14.5
Un skilled	3	0.9
Unemployment	1	0.3
Year of study		
First year MBBS	78	22.2
Second year MBBS	90	25.6
Third year MBBS	52	14.8
Final year MBBS	46	13.1
CRMI/internship	52	14.9
Postgraduate	33	9.4
Access to a private room/toilet facility at home or hostel	315	89.7

**Figure 1:**
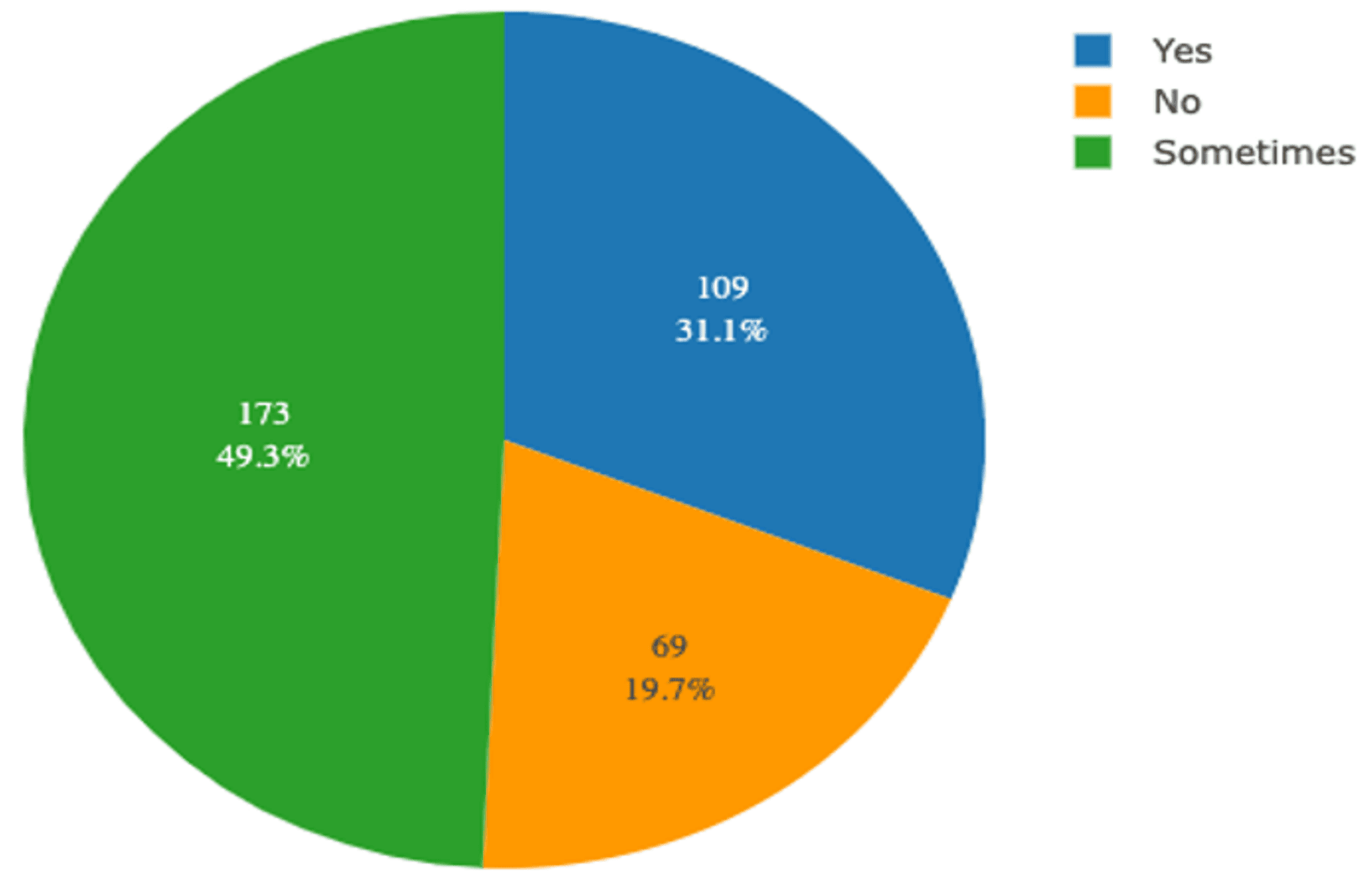
Distribution of study participants according to status of painful menstruation (N= 351)

The mean age of study participants at the time of menarche was 13 years (SD ±1.37), with the age of attaining menarche ranging from 9 to 13 years. Of the 351 study participants, 94 (26.8%) had irregular cycles, while the rest had normal menstrual cycles. The mean duration of the menstrual cycle among participants was 4.27 (±0.958) days, with the minimum duration being 1 day and the maximum 5 days. Of the 351 participants, 191 (54.4%) were day scholars and the remaining 160 (45.6%) were hostellers. Among the participants, 10 (2.8%) belonged to a family of 1-2 members, 295 (84%) to a family of 3-5 members, 40 (11.4%) to a family of 6-8 members, and 6 (1.7%) to a family of more than 8 members. The distribution of study participants according to the status of painful menstruation is depicted in Figure 1.

**Figure 2:**
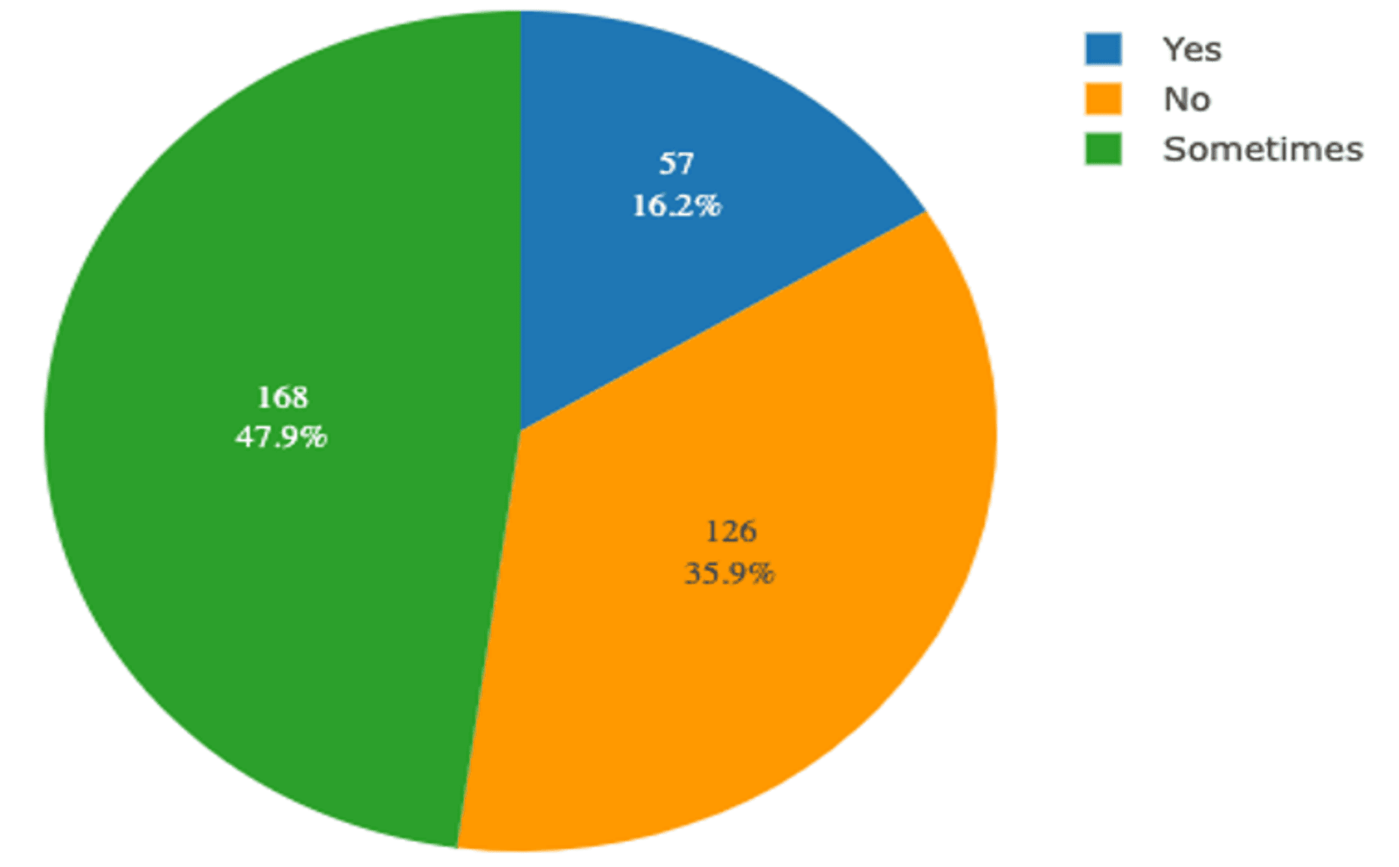
Distribution of study participants according to the effect of menstruation on their routine work/ studies (N=351)

**Figure 3:**
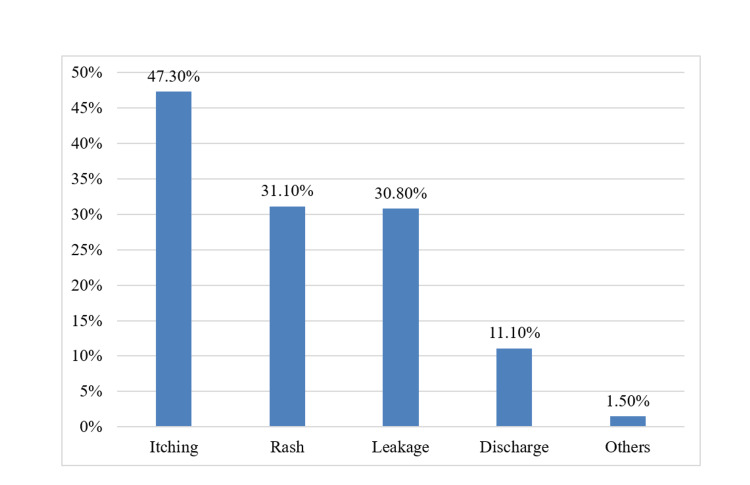
Symptoms associated with sanitary pad usage (N=331)

Among the study participants, approximately 93.7% used sanitary pads, 3.4% used menstrual cups, and 1.4% used tampons and reusable pads. Further details regarding the use of biodegradable products, symptoms experienced during usage, spending on products, and duration of product usage are presented in Table 2. Additionally, the distribution of study participants according to the effect of menstruation on their routine work or studies is shown in Figure 2. The various symptoms experienced by participants during sanitary pad usage are illustrated in Figure 3.

**Table 2:** Distribution of study participants according to menstrual hygiene practices (N=351)

Variables	Frequency	n (%)
Type of menstrual hygiene product used		
Sanitary pads	329	93.7
Menstrual cup	12	3.4
Tampons	5	1.4
Washable/reusable pads	5	1.4
Usage of biodegradable sanitary pads	142	43
Number of pads used per day during menstruation		
1 to 3 pads	172	50.9
4 to 6 pads	158	46.7
More than 6 pads	8	2.4
Presence of symptoms associated with pad usage (rash, itching, leakage, discharge, and others)	331	94.3
Spending on sanitary pads per cycle		
Less than Rs. 250	133	40.2
Rs. 250–500	157	47.4
Rs. 500–750	33	10.0
More than Rs. 750	8	2.4
Usage of a single sanitary pad for more than 6 hours		
Yes	108	32.1
Sometimes	144	42.9
No	84	25.0

**Figure 4:**
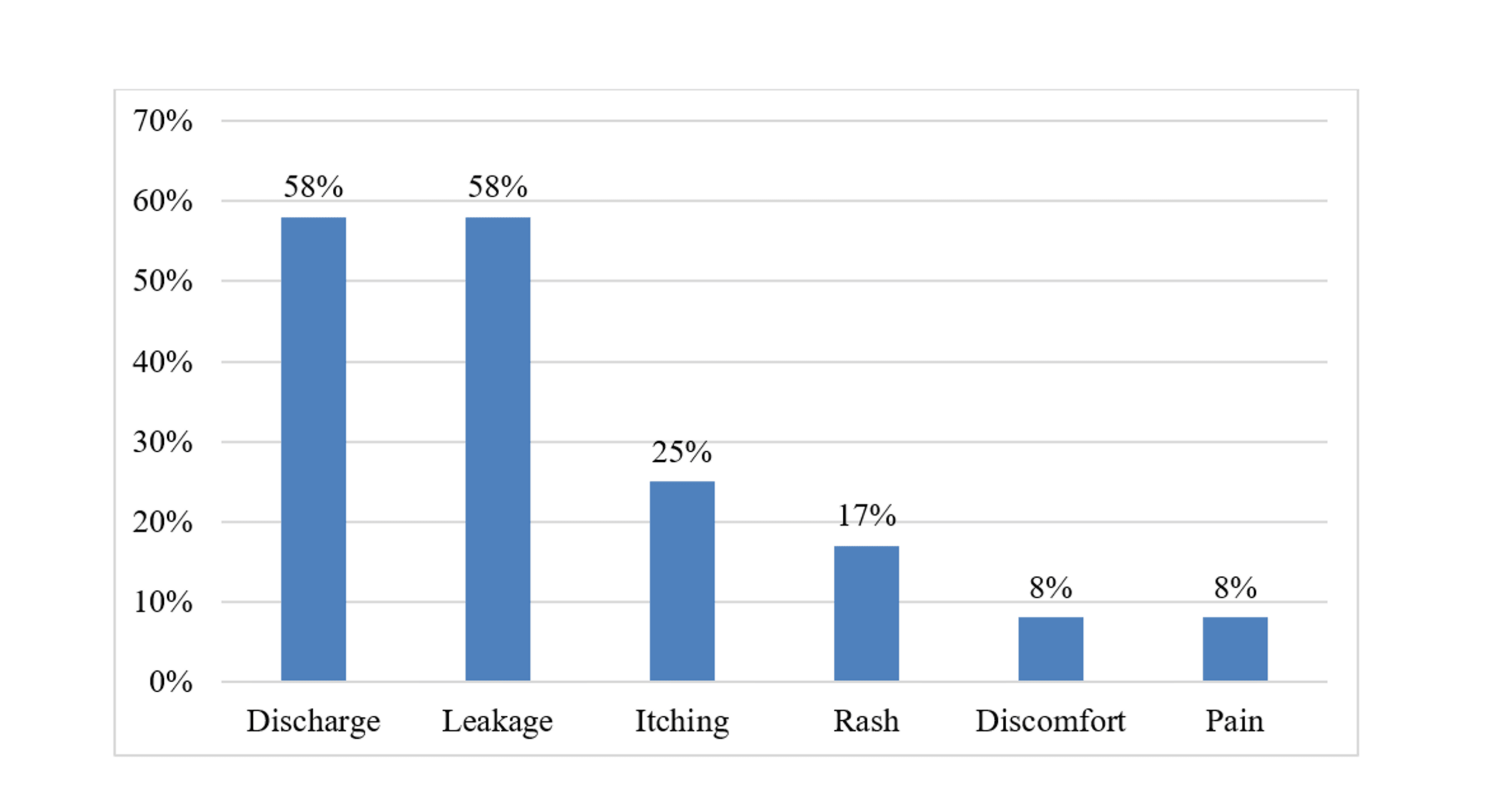
Symptoms associated with menstrual cup usage (n=12)

**Figure 5:**
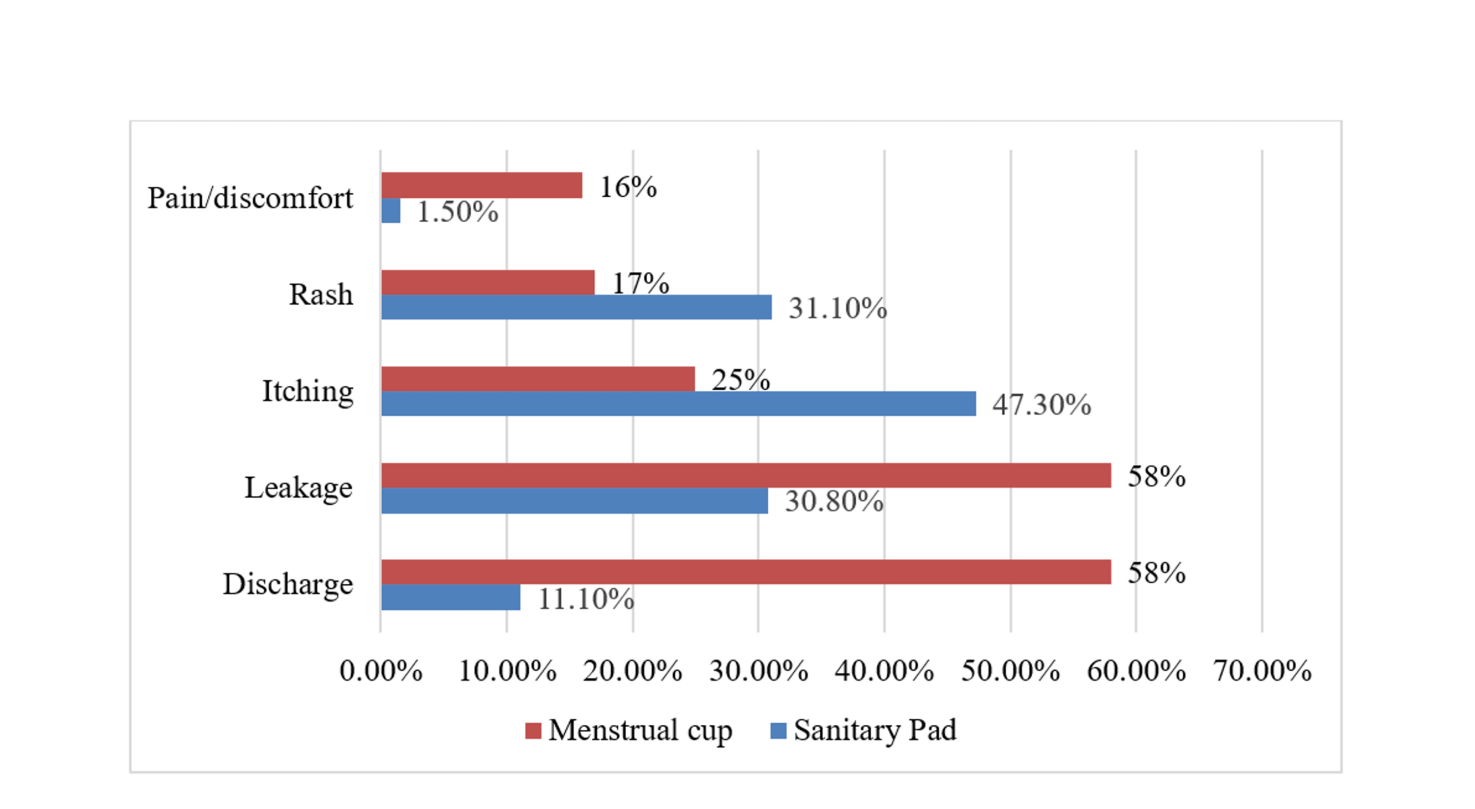
Comparison of symptoms associated with sanitary pad (n=351) and menstrual cup usage (n=12)

Among the 351 medical students, 179 (51%) reported washing their private parts more than three times per day during the menstrual cycle, 93 (26.5%) washed three times daily, 58 (16.5%) washed twice daily, 17 (4.8%) washed once daily, and the remaining 4 (1.1%) did not wash their private parts during the cycle. The prevalence of menstrual cup usage among the study participants was low, and the details are provided in Table 3. Our findings reveal that a majority of participants using menstrual cups reported complaints of discharge and leakage, which was much higher than those using sanitary pads. However, complaints of rashes and itching were relatively lower among menstrual cup users compared to sanitary pad users. It is noteworthy that the number of participants using menstrual cups in our study was limited, and hence the results should be interpreted with caution. Symptoms associated with the use of menstrual cups and sanitary pads are shown in Figure 4 and Figure 5, respectively.

**Table 3:** Distribution of study participants according to menstrual cup usage (n=12)

Variables	Frequency	n (%)
Duration of menstrual cup usage		
<1 year	7	58.3
>1 year	5	41.7
How much do you spend on menstrual cups every year?		
Less than Rs. 500	7	58.3
More than Rs. 500	5	41.7
How easy is it to insert a menstrual cup?		
Easy (scores 1 to 3)	7	58.3
Difficult (scores 4 and 5)	5	41.7
How easy is it to remove a menstrual cup?		
Easy	7	58.3
Difficult	5	41.7
How do you sanitize your menstrual cup after use?		
In boiling water	8	66.7
Washing with soap	2	16.6
Others	2	16.6
Quality of Life is good with menstrual cup usage	9	75
Presence of symptoms associated with pad usage	5	41.6

## Discussion

The mean age of the participants in our study was 21.4 years, with a standard deviation of ±2.59 years, and an age range spanning from 17 to 33 years. This relatively young participant group offers insights into menstrual hygiene practices among medical students who are generally in early adulthood, a crucial period for establishing sustainable hygiene habits. Additionally, the average age of menarche among these students was 13 years (SD ±1.37), aligning closely with menarcheal ages reported in several studies across India [10,11], indicating a consistent trend and possibly reflecting similar biological and environmental factors influencing the onset of menarche among young women in this region.

Our findings revealed that the majority of participants predominantly used sanitary pads over other menstrual hygiene products, such as tampons, menstrual cups, or reusable pads. This preference for sanitary pads is consistent with existing literature, including the study conducted by Anjana et al., which observed a similar trend among healthcare professionals from diverse sectors [11]. Moreover, a study by Shwetha Ballal et al. [12] reported menstrual cup usage of approximately 2.6% among participants, a figure that closely mirrors our findings. However, this percentage is lower than that reported in a study conducted in Kerala [11,13], where a higher usage of menstrual cups was observed among medical professionals. This discrepancy may be attributed to differences in levels of knowledge and awareness regarding alternative products, such as menstrual cups, among different professional groups, including healthcare workers and medical students.

In the studies by Adhikari et al. [14] and Juyal et al. [15], 86.36% of medical students used sanitary napkins as their primary absorbent material, while around 10.79% reported using cloth during menstruation, which are similar to the findings in our study [16]. This indicates that, despite awareness of other options, sanitary napkins remain the preferred choice for a significant majority, while a smaller percentage still relies on traditional practices such as using cloth. These insights underscore the need for increased education on alternative products that may offer improved comfort and hygiene.

Regarding adherence to recommended menstrual hygiene practices, 51% of our participants demonstrated good hygiene practices. While this is reasonably high, there remains scope for improvement to enhance health outcomes and reduce the risk of infections. Promoting greater adherence could significantly impact health, as better hygiene is associated with lower rates of menstrual-related infections and improved overall well-being. Another notable finding was the prevalence of symptoms associated with sanitary pad use, reported by nearly 58% of participants. This is lower than the prevalence reported in other studies, which ranged from 82% to 90% [17,18]. This variation may be attributed to differing levels of awareness about proper sanitary pad usage and hygiene maintenance among study populations.

Finally, we observed that the monthly expenditure on sanitary pads was below ₹250 for most participants, highlighting the relative affordability of this option compared to other menstrual hygiene products. This cost-effectiveness likely contributes to the continued widespread use of sanitary pads, as students often seek accessible and economical solutions to meet their hygiene needs.

Limitations

Potential biases, such as self-reported data and convenience sampling, remain critical for the generalizability of the study. Even though efforts were made to double-check the questionnaire, some degree of misinterpretation remains a possibility.

## Conclusions

Ensuring clean washroom facilities accessible for the staff alone and scheduling breaks for menstrual hygiene needs can help make menstrual hygiene among medical students and healthcare professionals more manageable. Both sanitary pads and menstrual cups have their own benefits and limitations. Their use depends on women’s personal preferences. When a woman uses a menstrual cup correctly, it can be a safe, cost-effective, and environmentally friendly alternative to pads. However, using a menstrual cup involves a learning curve, but with time and practice, a menstrual cup can become a woman’s preferred option. Promoting awareness about menstrual cups is crucial to encourage their adoption and empower individuals with a sustainable and effective menstrual hygiene product.
